# Double-Windows-Based Motion Recognition in Multi-Floor Buildings Assisted by a Built-In Barometer

**DOI:** 10.3390/s18041061

**Published:** 2018-04-01

**Authors:** Maolin Liu, Huaiyu Li, Yuan Wang, Fei Li, Xiuwan Chen

**Affiliations:** 1 Institute of Remote Sensing and GIS, Peking University, No. 5 Yiheyuan Road, Haidian District, Beijing 100871, China; maolin@pku.edu.cn (M.L.); pkuwangy@pku.edu.cn (Y.W.); gisor.lee@gmail.com (F.L.); xwchen@pku.edu.cn (X.C.); 2 China Ship Research and Development Academy, No. 2, Shuangquanbao, Chaoyang District, Beijing 100101, China

**Keywords:** motion recognition, barometer, double-windows, imbalanced data

## Abstract

Accelerometers, gyroscopes and magnetometers in smartphones are often used to recognize human motions. Since it is difficult to distinguish between vertical motions and horizontal motions in the data provided by these built-in sensors, the vertical motion recognition accuracy is relatively low. The emergence of a built-in barometer in smartphones improves the accuracy of motion recognition in the vertical direction. However, there is a lack of quantitative analysis and modelling of the barometer signals, which is the basis of barometer’s application to motion recognition, and a problem of imbalanced data also exists. This work focuses on using the barometers inside smartphones for vertical motion recognition in multi-floor buildings through modelling and feature extraction of pressure signals. A novel double-windows pressure feature extraction method, which adopts two sliding time windows of different length, is proposed to balance recognition accuracy and response time. Then, a random forest classifier correlation rule is further designed to weaken the impact of imbalanced data on recognition accuracy. The results demonstrate that the recognition accuracy can reach 95.05% when pressure features and the improved random forest classifier are adopted. Specifically, the recognition accuracy of the stair and elevator motions is significantly improved with enhanced response time. The proposed approach proves effective and accurate, providing a robust strategy for increasing accuracy of vertical motions.

## 1. Introduction

Motion recognition on smartphones is of great significance, and plays an important role in indoor and outdoor positioning [1], human activity recognition [2], remote health monitoring [3] and location-based services [4]. For indoor positioning, motion recognition is the basis of the pedestrian dead reckoning algorithm, which needs to estimate a user’s step length by identifying the motion state of the user and the attitude of the smartphone [5,6,7]. Equipped with a variety of sensors, smartphones are more attractive for mobile computing and awareness with ubiquitous and user-friendly superior features when compared to other devices. In addition, they do not disturb peoples’ normal activities.

Current research focuses on the utilization of a variety of built-in sensors of smartphones to identify motion states. These sensors include global positioning systems (GPS), accelerometers, gyroscopes, microphones, magnetometers, barometers, etc. [8]. The traditional motion recognition algorithms were only based on accelerometer data in the beginning. Nowadays, as more and more sensors are being embedded into smartphones, the most widely used approach is based on a combination of three sensors: accelerometer, gyroscope and magnetometer (AGM). The recognition accuracy can reach 90% for common motions [2,9,10], such as being static, walking, running, and so on. However, motion recognition in the vertical direction continues to be a challenge. The recognition accuracy for vertical motions is only 70%~80% [9,11,12], which is about 15% lower compared to motion recognition in the horizontal direction.

The emergence of smartphones’ built-in barometers brings an opportunity to improve the recognition accuracy of vertical motions. Although barometers were first integrated in smartphones to get coarse-grained altitude information for GPS positioning, many research studies have focused on their application in motion recognition. Google first embedded a barometer in the Galaxy Nexus in 2011 [3], Since then, more and more companies have imitated this design (e.g., Samsung Galaxy S series, Apple iPhone 6s, and Huawei Mate 8). Using the built-in barometer, the accuracy of the motion recognition in the vertical direction has been improved [13,14]. However, there is a lack of both quantitative analysis and modelling of the built-in barometer signals, as well as the theories of barometer application in multi-floor motion recognition. In Gu’s work [13], there is a delay in accurately recognizing the motions of going downstairs or upstairs when users transfer from walking state to going downstairs or upstairs. The problem is the same when users transfer from standing still to down-elevator or up-elevator. In addition, as people generally spend much more time on the same floor than moving between floors, more attention should be paid to an imbalanced data problem. In previous research, motion data were assumed balanced data in order to train classifiers. In fact, the frequency of some motions is much lower than that of other motions. Taking a regular scene as an example, people spend only a few minutes taking an elevator or climbing stairs, while spending tens of minutes, or even several hours moving at the same floor. Obviously imbalanced data will be generated in these situations.

The three main contributions of this work are summarized as follows: first of all, since the vertical speeds of the motions of climbing stairs and rising in an elevator are different, the corresponding pressure change rates also differ. With a single time window, it is difficult to simultaneously consider the two conditions, so the accuracy of distinguishing between the two motion states is not high. Based on the above considerations, a novel double-windows pressure feature extraction method based on the analysis of properties of smartphones’ built-in barometer signals is proposed, to balance the recognition accuracy and the response time. The random forest (RF) is an excellent classifier compared with other classifiers. However, if its samples are imbalanced, it is easier for majority categories to get more votes than minority categories when adopting the majority voting rule. Class voting distribution can reflect the classification result of samples to some extent. Therefore, a correlation rule for RF is designed to reduce the impacts of imbalanced data on classification accuracy. Furthermore, field experiments are conducted and the results validate the effectiveness and high accuracy of the proposed model and classification algorithm.

The rest of this paper is organized as follows: related research work concerning barometer-assisted multi-sensor fusion methods and imbalanced data is reviewed in Section 2. Section 3 focuses on the proposed double-windows pressure feature extraction method and the correlation rule for the RF classifier. The experiment, together with analysis of the results, is then arranged in Section 4. Finally, the research work in this paper and directions for future study are summarized in Section 5.

## 2. Related Work

The multi-sensor fusion motion recognition algorithm can be divided into four steps [9], namely sensor data sampling, data preprocessing, feature extraction and classification recognition. Current research focuses on the following three aspects: multi-sensor fusion, feature selection, and continuous motion recognition. In recent research, several sensors were combined to improve the recognition accuracy. As the number of built-in sensors increases, motion recognition algorithms are based primarily on the combination of AGM sensors, not merely based on the accelerometer [15,16]. At the same time, some other built-in sensors, such as the microphone [2], the barometer [2,13,17] and the camera [18], are also considered in some algorithms. What’s more, features are selected to balance the recognition accuracy and the amount of computation required. The recognition accuracy increases as more and more features are involved, including statistical features, time domain features, and frequency domain features, which dramatically increase the amount of computation. With the rapid development of deep learning, some researchers have conducted motion recognition by using a convolutional neural network [19,20]. However, these deep learning methods also cannot avoid a huge computational cost, which makes their use hard for real-time recognition, therefore, a balance of computation and accuracy is needed. Saeedi et al. [9] proposed a recognition algorithm using only four features that achieved 96.2% recognition accuracy. Khan et al. [2] selected the most relevant features from each sensor for each motion based on kernel discriminant analysis. This yielded recognition scenarios that are closer to reality, considering online continuous recognition instead of offline classification, so that the entire process can be run on a smartphone. Qian et al. [21] divided complex pedestrian motions into a series of fundamental motions, which can be easily recognized, and the conditional random field (CRF) algorithm is used to deal with the continuous recognition. In contrast, Liu et al. [22] adopted the Hidden Markov Model (HMM) algorithm. Other research hotspots include the universality of the recognition algorithm [23].

Despite these improvements, the recognition accuracy of vertical motions, such as going up or down stairs or taking elevators, is relatively lower than that of horizontal motions. Pei et al. [11,12] proposed a motion recognition algorithm based on AGM sensors with an overall recognition accuracy of 95.53% for eight common motions, but in contrast, only 77.78% for stair motions. Saeedi et al. [9] proposed a similar recognition for 13 common motions with 96.21% accuracy. Similarly, their stair motions recognition accuracy is only 70.15%.

The emergence of a built-in barometer brings an opportunity to improve the recognition accuracy of vertical motions. A barometer was integrated in smartphones for the purpose of getting coarse-grained altitude information for GPS positioning, and speeding up the GPS positioning. On this basis, the built-in barometer is further used to assist indoor positioning [24,25,26,27], altitude change monitoring [17] and motion recognition [13]. The applications are all based on the pressure-altitude correlation model [2,24,28,29], expressed as Equation (1):

(1) $$p_{abs}\left(h\right)=p_{0}\cdot {\left(1-\frac{0.0065\cdot h}{T_{0}}\right)}^{5.255}hPa$$

where *p_abs_*(*h*) is pressure, *h* is altitude, and *p*_0_ and *T*_0_ are the pressure reference and temperature reference, respectively. The reference conditions are usually set as 288.15 K (15 °C), where *p*_0_ = 1013.25 hPa, *T*_0_ = 288.15 K. However, the pressure is not only influenced by the height, but also by the environmental factors, such as weather, temperature, humidity, and wind speed [27,30]. Because of the limitations of natural conditions, most of the environmental factors wouldn’t change suddenly, except for the influence of human factors. For example, the sudden changes in pressure would be caused by opening or closing a door. In the design of modern barometer sensor, a temperature sensor was bundled into the barometric sensor chipset, for the purpose of compensating the errors in pressure reading caused by sudden changes of temperature [14]. Vanini et al. [14] conducted experiments from indoor to outdoor where the temperature difference was significant, to verify the effects of rapid temperature changes on pressure readings. The results showed that there were no significant changes in the values of barometric pressure. As the sudden changes could be eliminated by compensating mechanism of barometer, more attention should be paid to the effect of the gradual factors on atmospheric pressure. For the barometer, even if it is placed in a fixed location, the pressure values fluctuate noticeably during one day. More seriously, there are significant differences among measurements from different barometers [25,30,31]. Vanini et al. [31] found that the difference between two smartphones (Galaxy Nexus and Galaxy S3) under identical conditions can be larger than 1.7 hPa, which is equivalent to about 14.53 m.

Altitude estimation derived from Equation (1) cannot be directly used in motion recognition, as it is not very accurate. Therefore, research has turned to feature extraction from pressure data, in order to avoid the former problems. Khan et al. [2] selected the first, middle, and last samples in a 3.5 s window as pressure features to assist motion recognition. Bianchi et al. [32] used the pressure average deviation between former and latter 2 s windows to detect falling motion. Vanini et al. [31] monitored altitude changes to make use of pressure standard deviation. Gu et al. [13] proposed a pressure feature, called pressure derivative, to recognize seven common motions with an accelerator, and its accuracy reaches 90.7%. However, recognition accuracy of stair motions is only about 80%. What’s more, the length of the sliding window is set as long as 10 s. When a user takes an elevator from the 1st floor to the 3rd floor, the proposed algorithm recognizes the elevator motions until the user arrives at the 3rd floor and walks out of the elevator. From this perspective, the real-time performance of the algorithm needs to be improved.

As the problem of data imbalance is ubiquitous in daily motions, solving this problem is also an important way to improve the accuracy of motion recognition, however, few studies have been done on this aspect before. Class imbalance problems exist in various fields of classification or regression, such as text classification, speech recognition, medical diagnosis, anomaly detection and so on. There are basically two strategies to solve this problem: a data processing based strategy and a classifier based strategy. A data processing based strategy can change class distributions by over-sampling approaches or under-sampling approaches. Representative over-sampling approaches include easy ensemble [33], Neighborhood Cleaning (NCL) rule [34], Condensed Nearest Neighbor (CNN) rule [35], GSVM-RU [36] and OSS [37], while over-sampling approaches are represented by SMOTE [38], Borderline-SMOTE [39], GA-SVM [40] and so on. A classifier-based strategy solves imbalance problems by three methods, namely improving the classifier internally, cost-sensitive learning methods and recognition-based one-class learning methods. In the field of motion recognition, Wu et al. [41] designed the mixed-kernel based weighted extreme learning machine (MK-WELM) to reduce the influence of imbalance datasets problems. The mixed kernel method was proposed to reduce the influence of kernel selection on performance of extreme learning machine, and the cost sensitive method was utilized to deal with the imbalance problem [41]. Based on several machine learning methods, Zhang et al. [42] proposed a new inference framework, where guided under-sampling was employed to obtain balanced labelled subsets, in order to accommodate the imbalanced nature of mobile sensing data.

Through the above summary it can be observed that there is great potential for improving the performance of motion recognition assisted by a built-in barometer. The pressure-altitude model is set up under an ideal condition, while its direct altitude estimation contains errors due to the noise environment and hardware limitation. Existing algorithms lack considerations of some details that are easily neglected, such as the difference between stair motions and elevator motions. In addition, there are no succinct nor effective methods to solve the imbalanced data problem for motion recognition. In order to overcome these problems, a novel double-windows multi-floor motion recognition algorithm assisted by a built-in barometer, and a correlation rule for random forest are proposed in this work.

## 3. Methods

### 3.1. Pressure Properties Analysis and Modeling

The properties of pressure signals of a smartphone’s built-in barometer are the basis of its application to motion recognition. This work analyzes pressure signals and models their probability distribution. The parameter selection problems of stair and elevator motion recognition are analyzed at the same time.

#### 3.1.1. Pressure Signal Probability Distribution Model

Pressure signals are not stable, their variations are under the influence of temperature, humidity and wind speed. Even if a barometer and the user are strictly static, the pressure signals fluctuate significantly in one day. However, in a short period of time (several seconds), the signal can be considered as only containing random noise. Through the experiment and observation, which will be introduced in Section 4.2, it can be assumed that the noise obeys a Gaussian distribution. The pressure signal probability distribution can be expressed as Equation (2):

(2) $$p_{w}\sim N\left(\mu ,\sigma_{w}^{2}\right)$$

where *μ* is the average pressure, *σ_w_* is the pressure standard deviation. When staying static or moving on one floor (defined as H-motions), *μ* is stable and satisfies Equation (1). In contrast, *μ* varies as the altitude changes when moving in a vertical direction (defined as V-motions), such as going up or down stairs, or taking elevators. The derivative of Equation (1) with respect to *h* is shown in Equation (3):

(3) $$\frac{dp_{abs}}{dh}=-0.12\cdot {\left(1-\frac{0.0065h}{288.15}\right)}^{4.255}hPa/m$$

The average altitude at the plain region is about 200 m, and the heights of major buildings are below 100 m, as the ones above 100 m are defined as super-high building structures. When *h* in Equation (3) changes between 200 and 300 m, the pressure derivative is between −0.1177 and −0.1166 hPa/m. Consequently, it can be assumed that the variation of pressure is proportional to the vertical displacement, and the rate is −0.117 hPa/m.

Some constants can be derived from Equation (3). The common floor height is between 3.2 and 4.0 m so that the pressure difference between adjacent floors is between 0.3744 and 0.4680 hPa. The difference can reach 0.585 hPa owing to the fact that the floor heights of modern teaching buildings are around 4.5 or 5 m. The floor height in the experiment is about 4.6 m so that the pressure difference between adjacent floors is about 0.5382 hPa. The pressure difference between the 1st floor and the 4th floor is about 1.6146 hPa. Walking stairs between them lasts about 50 s, so that the average pressure change rate of stair motions is about 0.0323 hPa/s. For the elevator situation, the classic vertical speed of elevators is about 1.0 m/s so that the average pressure change rate is about 0.1170 hPa/s.

Overall, the pressure variation in multi-floor buildings can be divided into two parts, one is derived from altitude variation and the other is caused by Gaussian noise. In order to distinguish motions moving on one floor and between floors, the classification depends on features extracted from their two pressure time-series data. A pressure feature called pressure change rate (PCR) is analyzed in detail.

#### 3.1.2. PCR Probability Distribution Model

PCR is defined as the linear fitting slope of pressure time series data in a sliding time window. The time window length is labelled as *T*, and the pressure sample number in it is labelled as *N*. The pressure data obeys the Gaussian distribution expressed as Equation (2) so that PCR can be derived as Equation (4) according to the linear fitting algorithm:

(4) $$PCR=\frac{\sum_{i=1}^{N}t_{i}\cdot p_{i}-N\cdot \bar{t}\cdot \bar{p}}{\sum_{i=1}^{N}t_{i}^{2}-N\cdot {\left(\bar{t}\right)}^{2}}$$

where *p_i_* is the pressure value at epoch *t_i_*, $\bar{p}$ is the average pressure value in the sliding window, and $\bar{t}$ is corresponding average time value. Considering the continuous sampling rate of a barometer is approximately constant, we can assume it as a uniform sampling so that the *N^th^* sample is at epoch ti=iNT. Putting this expression into Equation (4), the PCR expression can be updated as Equation (5):

(5) $$PCR=\sum_{i=1}^{N}\frac{12\left(i-\frac{N+1}{2}\right)}{\left(N^{2}-1\right)\cdot T}\cdot p_{i}$$

**Figure 1:**
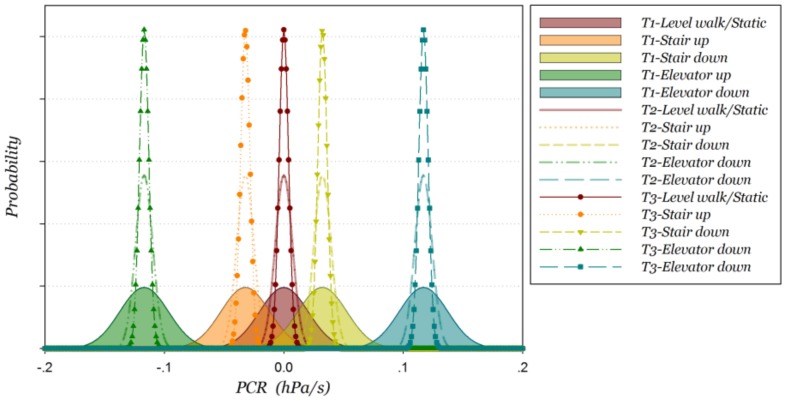
PCR probability distribution of different time-window lengths at different motion states.

**Figure 2:**
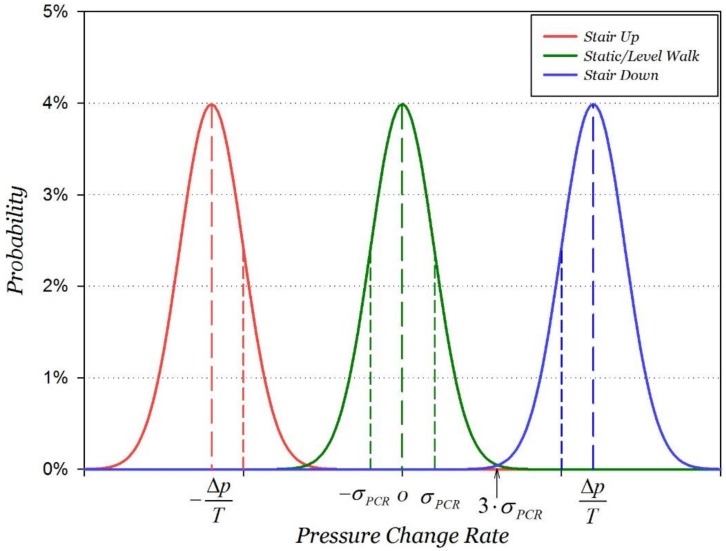
Distinguish H-motions and V-motions using PCR.

According to Gaussian distribution theory, the probability distribution of PCR also obeys the Gaussian distribution. In H-motions, the altitude remains stable so that pressure data within the sliding window obey the Gaussian distribution $N\left(0,\frac{12f\sigma_{w}^{2}}{(f^{2}T^{2}-1)T}\right)$, where *f* is the sampling frequency. The sampling frequency *f* and pressure standard deviation *σ_w_* are only related to the barometer hardware, therefore, the only parameter that affects the standard deviation of PCR is the length of sliding window *T.* We define $\eta \left(T\right)=\sqrt{\frac{12f}{\left(f^{2}N^{2}-1\right)T}}$ as the PCR fluctuation coefficient. In H-motions, the probability distribution of PCR obeys the Gaussian distribution $N\left(0,{\left(\eta \cdot \sigma_{w}\right)}^{2}\right)$. Similarly, N(ΔpT,(η⋅σw)2) is the Gaussian distribution of V-motions, where V*_p_* is the variance of pressure. As derived in Equation (3), average pressure change rate is about 0.0323 hPa/s for stair motions and 0.117 hPa/s for elevator motions. Using the standard deviation of PCR, the PCR probability distribution of different motion states is shown in Figure 1, and T1~T3 represent 1~3 times of unit sliding time window length. From the figure we can see that, the overlap of the PCR probability distribution between the motion states is gradually reduced as the length of the sliding time window gradually increases, which means the accuracy of motion states recognition will improve. The overlap of two PCR probability distributions should be as small as possible in order to distinguish between H-motions and V-motions. Usually three times the standard deviation is selected as the confidence interval of a Gaussian distribution so that two PCR probability distributions should obey Equation (6), as shown in Figure 2.

(6) 3⋅η⋅σwT≤|ΔpT−0|2

Specifically, the theoretical probability of recognition between stairs-motion and walking on the same floor can be expressed as Equation (7):

(7) F(T)=∫−PCRstair2PCRstair212π(η⋅σw)2e(x−0)22(η⋅σw)2dx

Obviously, the length of the time window cannot be infinitely long, and a longer time window means more time will be required for motion recognition. Vertical movement does not last too long, for example, it only takes 10 s to ascend from the 1st floor to the 4th floor by elevator. Consequently, a balance point needs to be found to make recognition accuracy high enough, while the length of the time-window is relatively short. For instance, when the sampling frequency *f* is 5.55 Hz and the pressure standard deviation *σ_w_* is 0.039 hPa, the standard deviation estimation of the probability distribution of PCR is expressed as follows:

(8) $$\sigma_{PCR}=\sqrt{\frac{12\cdot 5.55}{(5.55^{2}T^{2}-1)\cdot T}}\cdot 0.039=\frac{0.31827441}{\sqrt{(5.55^{2}T^{2}-1)\cdot T}}$$

**Figure 3:**
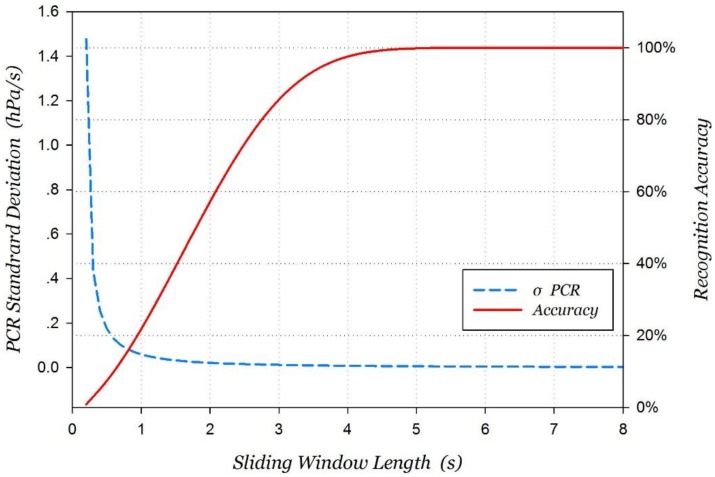
Recognition accuracy of the upper and lower moving states with sliding window length.

According to the model simulation, the curve map of the recognition accuracy of the upper and lower moving state with the length of the time window can be shown in Figure 3.

Usually, three times the standard deviation are chosen as the confidence interval of a Gaussian distribution. According to Equation (6), the optimal sliding window length for stairs motion recognition is *T* = 4.844 s by computer approximation calculation. Similarly, the optimal sliding window length for elevator motion is *T* = 1.435 s. In the real-world environment, a barometric signal is not stable, and it is influenced by environmental factors. Meanwhile, the shorter the sliding window length is, the easier the barometer is affected by the disturbance of the external environment. In a comprehensive analysis, the sliding window lengths used for stair motion recognition and elevator motion recognition are set 5 s and 2 s respectively.

As stairs motion and elevator motion can be distinguished from H-motions by different sliding windows, the remaining issue is to distinguish between elevator motion and stairs motion. According to the above analysis of vertical speeds, the vertical velocity of elevator is about 3.5 times higher than that of stairs motions under normal circumstances. Consequently, PCR plays a significant role in distinguishing between elevator motion and stairs motion. At the same time, the impact of accelerometer on the final results cannot be ignored. The accelerations of mobile phone are relatively stable in elevator, while in stairs state the acceleration fluctuation of each axis of the phone is relatively large. Therefore, the accelerometer also plays an important role in distinguishing between elevator motion and stairs motion.

### 3.2. Multi-Floor Motion Recognition

#### 3.2.1. Multi-Floor Motions Definition

The first step for motion recognition is to define motion states, however, until now they lacked a standard classification method until now. Ling et al. [15] proposed classifying six motion states, including static, standing with hand swinging, normal walking with holding the phone in hand, normal walking with hand swinging, fast walking, and turning. Bayat et al. [16] proposed a different classification standard, including slow walking, fast walking, running, stairs-up, stairs-down and dancing.

It should be noted that the attitudes of smartphones in motions, such as holding in hand and hand swinging, would greatly impact the performance of motion recognition. It should be carefully considered in the recognition algorithm, however, a standard classification method has not been built up. Qian et al. [43] proposed classifying four smartphone attitudes, including texting, calling, in-hand, in-pocket. Some more detailed attitudes, such as trouser pockets, shoulder bags, and belt enhancements [3] are taken into consideration. In contrast, Sara et al. [9] directly study the eight contexts combining motions and smartphone attitudes, including walking with phone close to ear, walking putting phone in pants pocket, walking with phone on the belt, walking with phone in a purse, walking with phone in a backpack, walking-swinging arm while device is in hand, stairs reading, and stationary texting.

This work focuses on an application scenario of indoor positioning and navigation in a modern multi-floor building so that motions are considered at only two smartphone attitudes: Hand-Using (HU) and Hand-Swing (HS). Because we want to verify the algorithm performances in distinguishing V-motions from H-motion, typical V-motions and H-motions of users are classified as one of six situations, including static, walking, stair-up, stair-down, elevator-up and elevator-down. Overall, the multi-floor motions in this paper are divided into nine kinds, listed in Table 1.

#### 3.2.2. Double-Windows Pressure Feature

H-motions and V-motions can be distinguished by their PCR feature, however, the preferred sliding window length is different. According to constants estimated in Section 3.1, the average PCR of stairs motions is 0.0323 hPa/s, and that of elevator motions is 0.117 hPa/s. Based on Equation (6), the required standard variation of PCR is about 0.005382 hPa/s, which is equivalent to a 5 s sliding window, with the purpose to distinguish stairs motions and H-motions. Similarly, 0.0195 hPa/s and a 2 s sliding window is for distinguishing elevator motions and stairs motions.

**Figure 4:**
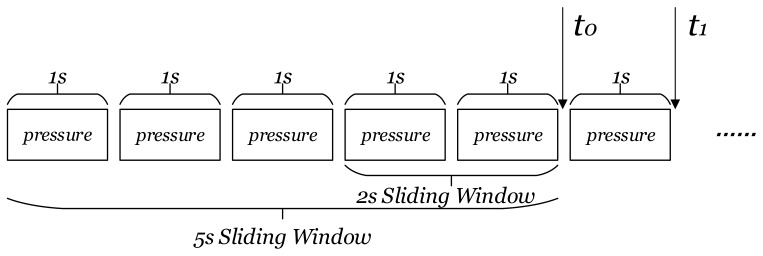
Double-windows pressure feature.

In brief, the work proposes a double-windows pressure feature to balance recognition accuracy and response time in multi-floor motion recognition. The two sliding windows, which are 5 s and 2 s respectively, are set up on the pressure data to extract features, and the sliding step length is 1 s, shown as Figure 4.

#### 3.2.3. Features and Sliding Window

Feature selection and the corresponding sliding window are key parts of the motion recognition performance. The proposed motion recognition algorithm fuses the features of AGM sensors in addition to the above proposed pressure features. There are many types of AGM sensor features, such as time domain, frequency domain, time-frequency domain, heuristic and domain-specific [9]. There are no theoretical guidelines that suggest the appropriate features to use in a specific classification situation. Among every recognition algorithm, there are significant differences in the selected features. The differences are not only the types of features, but also the number of features. Bayat et al. [16] adopted 24 features, and Pei et al. [11,12] employed 27 features. By contrast, Saeedi et al. [9] adopted only four features. Considering the real-time requirement, the proposed motion recognition algorithm only adopts time domain features, which include 15 features listed in Table 2.

The length of the sliding window determines the balance between recognition accuracy and response time. The shorter the sliding window, the faster the system response and the better the user experience [43]. In contrast, if the sensor data is richer, the recognition accuracy would be theoretically higher. Saeedi et al. [9] concluded following an experiment that sensor data within 2 s contains sufficient features to represent each motions. As a result the length of the sliding window is usually selected as 2 s in many research studies [3,9]. Other choices include 2.56 s [23] and 3 s [2]. The overlap of continuous sliding windows can improve the output frequency, and the identical approaches set the overlap percentage as 50% [3,9,22,23]. Based on the researches discussed above, the length of the sliding window is set as 2 s with 50% overlap and 5 s with 80% overlap in this work. This means that the algorithm can output results every second.

### 3.3. Improved Random Forest Classifier for Imbalanced Data

The problem of imbalanced data is ubiquitous in motion recognition. In this part, the RF classifier is chosen to solve this problem. RF is a classification method integrating multiple decision trees. Each tree uses a random subset of the data for independent training [44]. The execution of RF is as follows: (1) Selecting the training set; K decision trees are built by sampling K times on the dataset. (2) Construction of the RF model; it is assumed that the training data set has M attributes, from which $\sqrt{M}$ attributes are randomly selected as the classification attribute sets. Then, Classification and Regression Tree (CART) is used to construct a single decision tree, which does not restrict the generation of each decision tree, and does not do any pruning. (3) Vote; the random forest classifier makes decisions by the greedy method, and uses K decision trees to classify certain data. It classifies the category that gets the most votes as the final output result of the random forest:

(9) $$OOBErr(RF)=1-\frac{\sum_{i}^{N}\left(o_{i}|v_{o_{i}}^{RF}=l_{o_{i}}\right)}{N}$$

RF improves the prediction accuracy by cross validation between decision trees of random samples, and effectively solves the problem of overfitting [45], without increasing the computational complexity. It evaluates the reliability of the model by out-of-bag error (OOBErr) (Equation (9)). In the bagging process of RF, each decision tree is constructed by re-sampling the original data feature set. For each sampling, several objects are not chosen as samples, and then they form the control group data set. Therefore, RF doesn’t need an extra portion of data for cross validation, and the OOBErr is the unbiased estimate of prediction error [46].

The RF model is not affected by imbalanced samples in the training stage, however, the impact of imbalanced data cannot be alleviated by the majority voting rule in the classification stage. In brief, the number of each sample category varies greatly. For instance, the number of walking samples is larger than that of stair samples. However, all categories of samples affect the selection of feature sets in the training process of random forests. In order to make the biggest decreasing amplitude of the Gini coefficient in the feature node, target objects are usually identified as the category which contains a large number of samples. For RF, the classification result is determined by the voting distribution $\left(n_{1},n_{2},\ldots ,n_{s}\right)$ with $\sum_{i=1}^{s}n_{i}=N$, where *S* is the number of categories, *N* is the number of decision trees, and *n_i_* represents the number of decision tree which vote for the *i^th^* category. The simple majority voting rules [47] determine the object classification result according to the $\max(n_{1},n_{2},\ldots ,n_{s})$. However, if the samples are imbalanced, it is easier for majority categories to get more votes than minority categories. Once the votes for any category are much larger or smaller than it deserves, misclassifications will occur.

Fortunately, the class voting distribution can reflect the classification result of samples to some extent, and the voting distribution of imbalanced samples is available, though the vote number for some categories are affected by imbalanced data. Let pi=niN be the probability of the *i^th^* category, then $prob(o)=(p_{1},p_{2},\ldots ,p_{s})$ is the vote probabilistic distribution of sample *o*. Representative distribution of each category is the average of reliable distributions, which are top 5% in probabilistic distributions of each category. The vote probabilistic distribution of an unclassified sample is compared with the representative distributions of *S* categories, and the category with the largest correlation coefficient (Equation (10)) is the classification result of the sample:

(10) $$category(i)=\frac{Cov[prob(o),prob(o_{i})]}{\sqrt{Var[prob(o)]Var[prob(o_{i})]}}$$

where *prob*(*o_i_*) is the vote representative distribution of the *i^th^* category. For RF, its time complexity [48] is *O*(*N_trees_M_features_n_samples_*log(*n_samples_*)),where *N_trees_* is the number of decision trees, *M_features_* is the number of features, and *n_sample_s* is the number of samples. The improved RF needs to additionally compute the probability of each category and the correlation coefficient, and their time complexity is *O*(*N_trees_*). Compared with the original RF, the improved RF shows negligible time complexity changes. According to actual testing, the time consumption differences of the above two kinds of RF algorithms are so small that they can be ignored in practical applications.

## 4. Experiment Evaluation

### 4.1. Experiment Set Up and Data Sample

**Figure 5:**
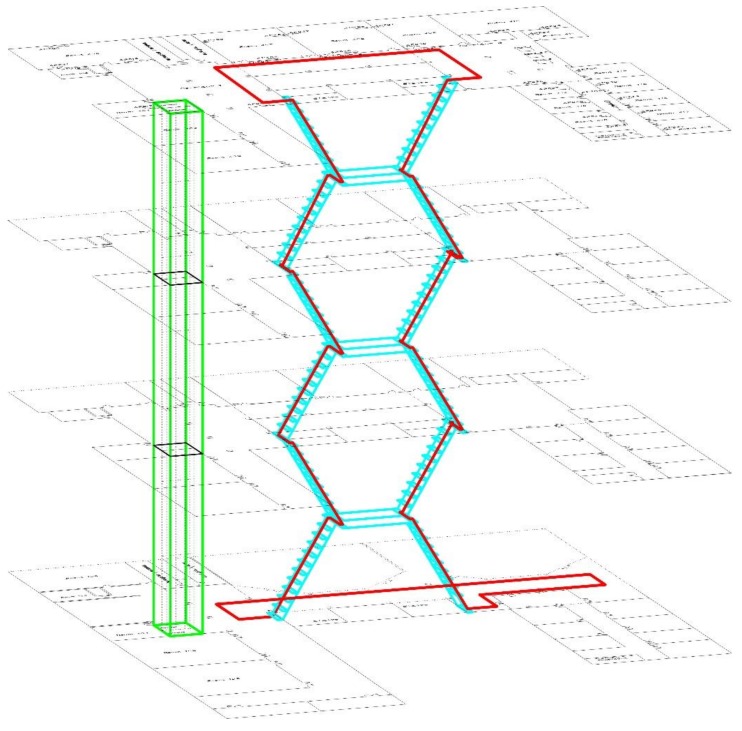
The three-dimensional layout of experiment region and movement path.

The experiment was conducted at the science teaching building at Peking University, which is a modern 4-floor building. The 3-dimensional layout of the experiment region and movement path is shown as Figure 5, where the parts marked by blue lines are stairs (including corner platform), the parts marked by green lines are elevators, and the red lines refer to testing movement paths. Two smartphones were used in the experiment, including a Samsung Galaxy S4 and a Google Galaxy Nexus. The inner barometers of the two smartphones are BPM182 [49] and BPM180 [50], respectively. The measuring range, relative pressure measuring accuracy and pressure noise value of the two barometers are the same, namely 300~1100 hPa, −0.12 hPa~+0.12 hPa and 0.05 hPa. However, the absolute pressure measuring accuracy is different, the former is −1.0 hPa~+1.0 hPa [3] and the latter is −4.0 hPa~+2.0 hPa [50].

There were 10 testing users, including six males and four females. According to the definition of multi-floor motions, the data sample phase was divided into the following three parts:

(1) Keep static with the smartphone held in hand for 2 min.
(2) Walk a lap along the movement path shown in Figure 5 at a normal pace. The whole journey needs about 10 min, including 2 min of stair-up, 2 min of stair-down, and 6 min of moving on one floor. The attitudes of smartphones are divided as hand-using and hand-swing.
(3) Take elevator from the 1st floor to the 4th floor for four times without considering the pause of the elevator. This takes about 4 min.

The data sample application is self-developed based on the Android operating system. The four sensors are set at the fastest sample frequency during sampling, where the sample mode was set as fastest in the Android API.

### 4.2. Pressure Model Validation

#### 4.2.1. Pressure Signal Characteristic Analysis

**Figure 6:**
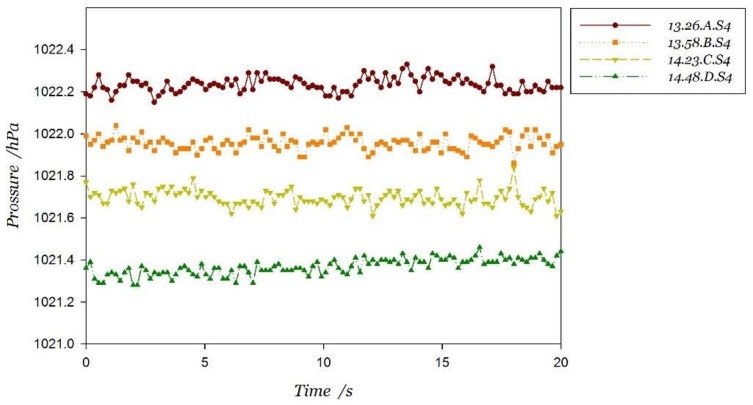

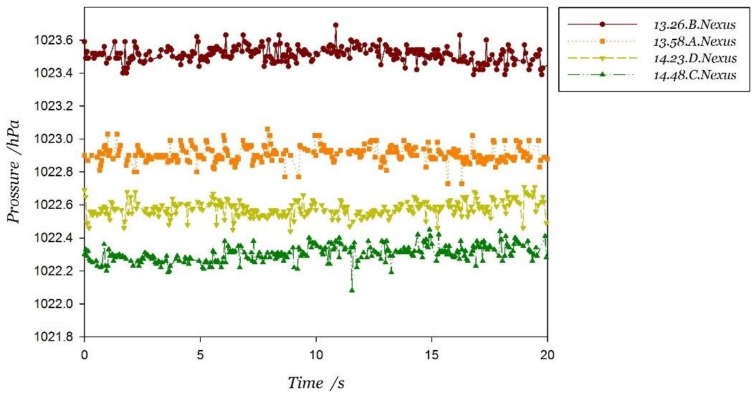
Air pressure data measured by two smartphones in stationary state.

In the static state, the air pressure data measured by two intelligent mobile terminals is shown in Figure 6. There are eight sets of pressure data, which are measured in four periods of time at the same location.

It can be found from the analysis of measurement results that: (1) The mean change in barometric pressure could be more than 1 hPa if the time interval is over 1 h (13:26–14:48). However, the measured values are relatively stable in a short period of time, fluctuating within the range of 0.2 hPa, which confirms the analysis in Section 3.1. The elevation estimated directly from the model of pressure and elevation probably contains some errors, which could reach 8.55 m in an hour, almost the height of two stories. (2) The measurement values of different barometers are different and the difference between observed values are not stable. As is shown in Table 3, the differences of measured values are 1.241 hPa, 0.936 hPa, 0.919 hPa and 0.951 hPa in four periods of time. Therefore, it is not feasible to estimate elevation by a differential pressure reference station owing to the instability of different barometers’ measurement differences.

**Figure 7:**
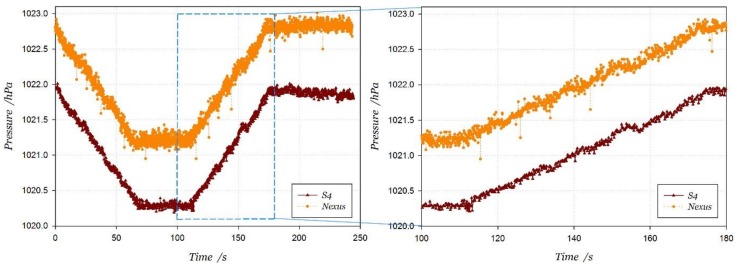
Air pressure data measured by two smartphones in Stair-up and Stair-down.

The built-in barometer signal comparison between two smartphones in stair motions is shown in Figure 7. It can be found that the pressure changes of different built-in barometers are consistent, which verifies that the relative change in the pressure measurements is not affected by hardware and can be applied to different built-in barometers.

**Figure 8:**
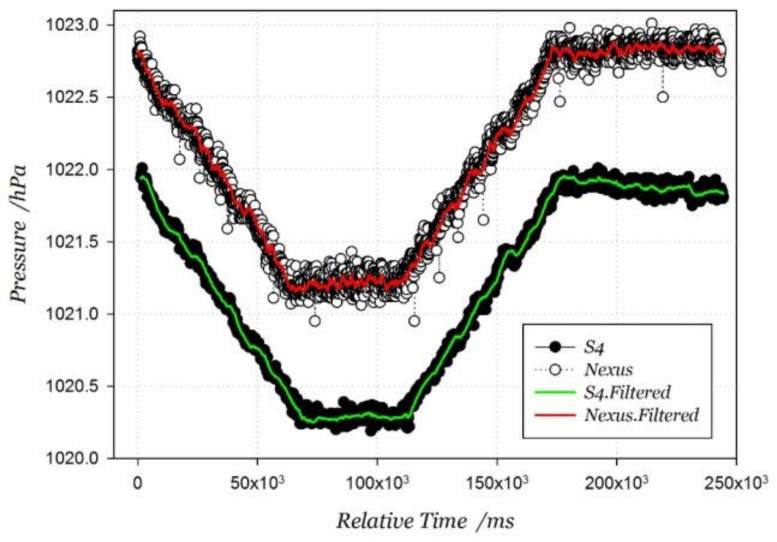
Pressure signals from two smartphones in stair motions.

In order to further improve the air pressure estimation accuracy, a double exponential smoothing filter is adopted to smooth the pressure data. The data before and after filtering are shown in Figure 8, where the label ‘Filtered’ in the legend represents the pressure data filtered by double exponential smoothing. The filtering algorithm is expressed as Equation (11), where *x_t_* is the original data at epoch *t*. *s_t_* is the smoothed value at epoch *t*, and *b_t_* is the best estimate of the trend. The filter has two adjusting factors, including data smoothing factor α and trend smoothing factor *γ*. Their value range is set between 0 and 1, and the empirical value is set 0.1 and 0.03, respectively. From the pressure signal measurements, there are obvious fluctuations even if users move on the same floor. The signal difference between two smartphones remains about 0.9 hPa, which is equivalent to around 7.7 m at altitude. It validates the conclusion that the altitude estimation from the pressure-altitude model is unreliable. However, the pressure variation due to stair motions is similar between the two smartphones:

(11) $$\begin{array}{l} s_{t}=\alpha x_{t}+\left(1-\alpha \right)\left(s_{t-1}+b_{t-1}\right)\\ b_{t}=\gamma \left(s_{t}-s_{t-1}\right)+\left(1-\gamma \right)b_{t-1} \end{array}$$

#### 4.2.2. Pressure Signal Probability Distribution Validation

**Figure 9:**
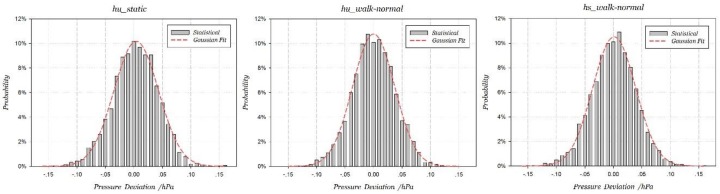
Gaussian fitted curves of pressure signals in three H-motions.

Then we statistically analyze the pressure signals in H-motions, including staying static or moving on the same floor with hand-using or hand-swing attitudes. For the Galaxy S4, the sample numbers in three motions are respectively 2775, 2517 and 2622, with an average sampling frequency of 5.55 Hz. The pressure standard deviation and Gaussian fitting correlation coefficient in three motions are listed in Table 4. The average pressure standard deviation is about 0.038 hPa, and the Gaussian fitting correlation coefficients in three H-motions are all larger than 0.9965. The fitted pressure signal probability distributions are shown as Figure 9.

Overall, the pressure signals in H-motions are correctly treated as obeying Gaussian distribution, and altitudes of the smartphone have no effect on the pressure signal probability distribution. Similarly, the average pressure standard deviation of Galaxy Nexus is about 0.0525 hPa.

#### 4.2.3. PCR Probability Distribution Validation

##### Different Sliding Window Length Experiments 

**Figure 10:**
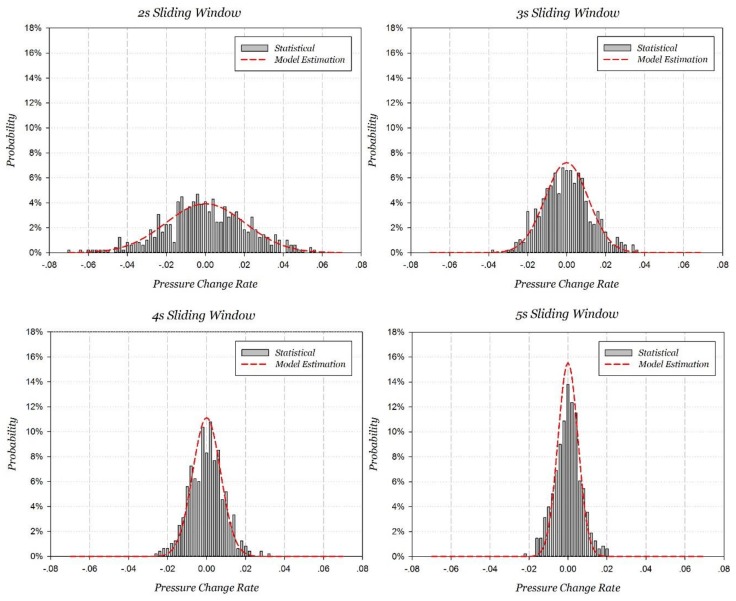
Statistical PCR probability distribution and model estimation at static with different lengths of sliding windows.

After changing the length of the sliding window from 2 to 5 s, PCR and its probability distribution were statistically analyzed. In order to eliminate other interference factors, the concentrated motion was only stationary state. The PCR standard deviations estimated in Section 3.2.2 and their corresponding correlation coefficients are listed in Table 5. The model estimated PCR probability distributions compared with the statistical ones are shown in Figure 10. The results show that the model estimation exhibits a significant positive correlation with the measured statistics, and the correlation coefficients are all above 0.935 at different lengths of the sliding window. Specially, the PCR standard deviation at 2 s and 5 s sliding window are 0.0203579 hPa and 0.0051326 hPa, respectively, which coincide with the double-windows set in Section 3.1.2.

##### Validation in Different Motions

**Figure 11:**
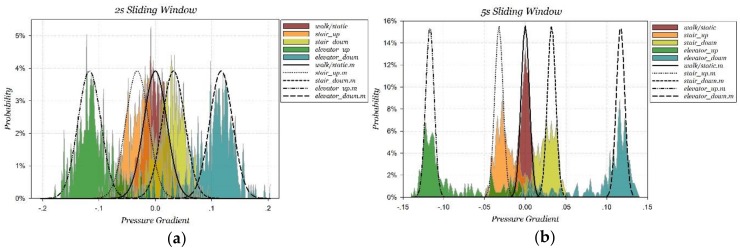
Statistical PCR probability distributions and model estimation at different motions. (**a**) 2 s sliding window, (**b**) 5 s sliding window.

The PCR features are extracted within 2 s and 5 s sliding windows in different motions, and are calculated for statistical probability distributions. Pressure signals in nine multi-floor motions can be divided into five classes, including walking/static, stair-up, stair-down, elevator-up and elevator-down. Using this classification, the PCR probability distributions within 2 s and 5 s sliding windows are shown as Figure 11. The corresponding correlation coefficients are listed as Table 6.

The experimental results show that the model-estimated PCR probability distributions in H-motions is accurate, where correlation coefficients are all above 0.966. In contrast, the model in V-motions has shifted observably, and all correlation coefficients are below 0.9 through the primary component while the distribution center estimates are accurate. There are three reasons leading to the misestimation:

- There are stair corner platforms during stair motions. There are five stair corner platforms among the stair motions from the 1st floor to the 4th floor, and each one takes 2~3 s by walking. The motion context during this period of time belongs to H-motions strictly. For the purpose of continuous motion recognition, motions during stair corner platforms are recognized as stair motions instead of H-motions in our experiments
- There are braking processes when taking the elevators so that the PCRs could decrease to zero. Our experiments were carried out at noon, during which time a lot of people entered or exited the elevator. The temporary static conditions caused by other passengers getting in and out of elevators would decrease the accuracy of elevator motion recognition.
- Data sampling experience continuous motions so that there are switching processes between different motions.

To enhance the applicability of algorithms, the three scenarios above need to be considered in our methods. As the motion states of users are generally smooth and continuous, which means that users do not intermittently change their motion states and each motion state lasts for a certain period of time, historical features [13] could be adopted in the recognition algorithm. The basic idea of historical features is to make use of the state history information and the characteristics of user’s motions to eliminate the jump phenomenon that the classifier reports varying motion states when the user is in the same state. It provides the potential to solve the problems of stair corner platforms and brief stop of elevator.

In conclusion, the PCR and its probability distribution model is correctly validated. It is feasible to distinguish elevator motions from H-motions when adopting a 2 s sliding window, and to distinguish stair motions from H-motions when adopting a 5 s sliding window.

### 4.3. Multi-Floor Motions Recognition Validation

#### 4.3.1. Recognition Performance Evaluation

Using the features extracted from the 4 built-in sensors, which are listed in Table 2, multi-floor motions can be recognized based on machine learning theories. The work adopts 5 commonly used machine learning algorithms, including Random Forest (RF), J48 Decision Tree (DT), Artificial Neural Network (ANN), Support Vector Machines (SVM) and Naive Bayes (NB). They are all implemented by the Weka toolkit [51] developed by Professor Witten [52], the university of Waikato, New Zealand. The 10-folds cross-validation is adopted, and the recognition accuracy of each multi-floor motion is compared in Table 7, where PCR2 and PCR5 represent the algorithm involving pressure features with 2 and 5 s sliding window respectively, and ‘Without P’ referring to none for pressure features. The result demonstrates two important conclusions. On the one hand, the recognition accuracy of algorithms involving the pressure features is significantly improved, and that of algorithms using RF can reach 92.58%. On the other hand, the recognition accuracy of algorithms based on double-windows pressure features is better than single-window approaches.

From Table 7, we can see that the recognition performance using RF and ANN is better than the other algorithms. Taking RF as an example, the real-time recognition accuracy of each motion using RF is compared in Table 8. All these can be easily run on a current smartphone with corresponding sensors.

The detailed recognition result further shows that different length of sliding window on pressure data have different effects on multi-floor motions, and the double-windows-based approach has the best performance. It means the recognition of elevator motions and stair motions cost only 2 s and 5 s, which is acceptable in most of the application scenarios.

#### 4.3.2. Improved Random Forest Validation

As observed in the experiments above, the performance of the RF algorithm is relatively good compared with several other machine learning algorithms. The motion recognition of RF is effectively improved by the double-windows pressure features, however, the performance of RF is still affected by imbalanced data. To further analyze the impact of imbalanced data, 4651 samples were used to train the RF model, and 1901 samples are used to test the accuracy of the model. What’s more, the category proportions of training samples and testing samples are approximately the same. The RF model contains 200 decision trees, each of which has one vote, and the classification result is the category with the most votes. For testing samples, the confusion matrix of the RF model is shown in Table 9, and the overall accuracy is 92.58%.

From the confusion matrix we know that: (1) Misclassification is serious between stair-motions and walk-motions. One reason is that features of the two kinds of motions are somewhat similar, and the other reason which should not be ignored is that samples of walk-motions are 2~3 times larger than that of stair-motions. In real life, the difference in sample numbers may be greater, for people generally spend much more time on the same floor than moving between floors. (2) The number of samples which are classified as the majority category is larger than the actual number, and for minority categories, the classification result is just the opposite. It is worth noting that the number of elevator-motions is small, but it is scarcely affected by the imbalanced problem. One possible reason for this is that the features of the elevator-motions are easily distinguishable, which also indicates the effectiveness of double-windows pressure feature on the secondary side.

**Figure 12:**
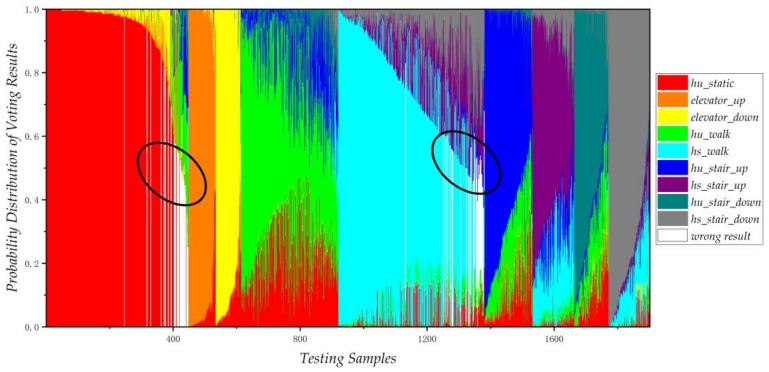
Voting proportional distribution of random forest.

Voting results of 1901 testing samples are presented using a proportional histogram in Figure 12. The horizontal axis represents testing samples, and the vertical axis represents the vote probability distribution of the random forest. Each vertical line stands for voting distribution of a testing sample, while the length of the different color line stands for its voting percentage. The samples containing the white line means they are misclassified, and the figure only shows the samples which are misclassified as hu-static and hs-walk.

For motion recognition, the data of RF is imbalanced due to variations in people’s daily behavior, and the characteristic difference of different motions varies significantly. There may be some bias in the categories that are determined by the most voting rules. Taking the cyan area (hs-walk) as an example, 32 samples (white line) of the other categories are recognized as hu-walk, and 13 hu-walk samples are classified as other objects. What’s more, from the concentration of white lines, it can be seen that the lower the voting rate of the sample (black line ellipse), the more likely it is to be misclassified.

The improved RF algorithm introduced in Section 3.3 recognizes samples by the distribution correlation coefficient, rather than the majority voting rule. Table 10 shows the confusion matrix of the improved RF, and the overall recognition accuracy reaches to 95.05%. What’s more, the number of hu-stair misclassified as hu-walk and the number of hs-stair misclassified as hs-walk has both been reduced, which fully illustrates the effectiveness of the improved RF algorithm.

## 5. Conclusions and Future Work

The paper focuses on the motion recognition problems during multi-floor positioning and navigation, and uses the novel built-in barometers in smartphones to improve the recognition accuracy of multi-floor motions, especially that of motions containing vertical movement. The advantages of the proposed methodology are mainly manifested in two aspects. On the one hand, it is about the excellent performance of recognizing stair and elevator motions. The double-windows strategy was established by analyzing pressure characteristic of stair motions and elevator motions. It can not only improve the recognition accuracy of vertical direction, but also shorten the response time due to that it can capture features within a relatively short period of time. In addition, the methodology shows great robustness. The problem of imbalanced data, which exists in the previous methodologies, is effectively solved by this method. In this paper, the pressure signals and pressure change rate were analyzed in detail. Through a mathematical model derivation, we further proposed the PCR probability distribution. Then we theoretically analyzed the length problem of sliding window for different multi-floor motions. In order to balance the recognition accuracy and response time, we proposed a novel double-windows. Furthermore, an improved random forest algorithm was proposed to deal with the imbalanced data problem. Finally, field experiments were conducted based on common machine learning algorithms, and the result clearly validated the outperformance of the proposed methods. The multi-floor motion recognition using the proposed pressure feature was significantly improved, and the real-time recognition accuracy reached 92.58% for nine multi-floor motions based on the random forest algorithm. After changing the voting rule, the accuracy further increased to 95.05%, and all this could be run on a current smartphone with corresponding sensors.

Certainly, there is still room for improvement in the proposed algorithm. Future work includes time-validity and universality improvement. On one hand, it is of practical value to run the motion recognition algorithm completely on a smartphone so that the complexity and resource consumption of the proposed algorithm are significantly limited. Though the proposed algorithm has avoided the complex frequency domain features, the contribution of each used feature can be further analyzed so that the computation load can be further decreased on the premise that the recognition accuracy is acceptable. In addition to the computational load, battery consumption is also an important issue which needs to be considered carefully. As we have adopted multiple sensors including accelerator, gyroscope, magnetometer and barometer, our approach isn’t ideal for electricity consumption. A possible solution to reduce power consumption is adaptive sensor selection. This means that various sensors are turned on and off in real time in an adaptive way for energy-efficient activity recognition. On the other hand, the tested users and movement path is limited in the experiment not considering the random situation arising in daily usage and personalization. All these are topics worthy of further study.

## Figures and Tables

**Table 1:** The definition of multi-floor motions.

No.	Motions of User	Smartphone Attitude
1	Static	Hand-Using
2	Elevator-Up	Hand-Using
3	Elevator-Down	Hand-Using
4–5	Walking	Hand-Using/Hand-Swing
6–7	Stair-Up	Hand-Using/Hand-Swing
8–9	Stair-Down	Hand-Using/Hand-Swing

**Table 2:** Multi-sensor features used in motion recognition.

No.	Sensor	Feature	Note
1	Accelerator	accXMean	*X*-axis average acceleration
2		accYMean	*Y*-axis average acceleration
3		accZMean	*Z*-axis average acceleration
4		accXSD	*X*-axis acceleration standard deviation
5		accYSD	*Y*-axis acceleration standard deviation
6		accZSD	*Z*-axis acceleration standard deviation
7		accRSD	Result acceleration standard deviation
8		accRMCR	Result acceleration Mean Crossing Rate
9	Gyroscope	gyrRMean	Average result angular acceleration
10		gyrRSD	Result angular acceleration standard deviation
11	Magnetometer	rollSD	Roll angular standard deviation
12	Barometer	preSD_2	Pressure standard deviation (two seconds)
13		preCR_2	PCR (two seconds)
14		preSD_5	Pressure standard deviation (five seconds)
15		preCR_5	PCR (five seconds)

**Table 3:** The mean and the difference of the pressure (hPa) measured by mobile terminals.

Time	13:26	13:58	14:23	14:48
S4	1022.252	1021.952	1021.682	1021.363
Nexus	1023.493	1022.888	1022.601	1022.314
Difference	1.241	0.936	0.919	0.951

**Table 4:** Statistical analysis of pressure signals in three H-motions.

	hu_static	hu_walk	hs_walk
Sample Number	2775	2517	2622
Standard Deviation	0.03954	0.03749	0.03874
Correlation Coefficient	0.9965	0.9970	0.9979

**Table 5:** PCR probability distribution model estimation and correlation coefficient.

F(Hz)	N	α(N)	$\sigma_{w}$(hPa)	T(s)	Estimated SD (hPa/s)	Correlation Coefficient
5.55	11.10	1.043996	0.039	2	0.0203579	0.935175
5.55	16.65	0.850488	0.039	3	0.0110563	0.965057
5.55	22.20	0.735962	0.039	4	0.0071756	0.962634
5.55	27.75	0.658023	0.039	5	0.0051326	0.981629

**Table 6:** Correlation coefficients in different motions with 2 and 5 s sliding window.

Window	Walking/Static	Stair-Up	Stair-Down	Elevator-Up	Elevator-Down
2 s	0.966579	0.876182	0.891987	0.862608	0.873213
5 s	0.980465	0.776383	0.765242	0.84696	0.847342

**Table 7:** Multi-floor motion recognition accuracy comparison using different algorithm.

Algorithm	RF	DT	ANN	SVM	NB
Without P	83.31%	75.64%	77.36%	78.23%	74.80%
PCR2	91.35%	87.43%	92.61%	89.35%	88.31%
PCR5	91.03%	86.55%	91.13%	87.69%	87.56%
PCR2&5	92.58%	89.35%	92.47%	91.59%	89.72%

**Table 8:** Each motion recognition accuracy using RF.

Class	Without P	PCR2	PCR5	PCR2/5
hu_static	79.8%	92.3%	91.1%	92.5%
hu_elevator-up	73.6%	91.4%	88.2%	93.7%
hu_elevator-down	79.7%	93.7%	93.3%	92.9%
hu_walk	89.3%	86.3%	90.1%	90.2%
hs_walk	88.4%	91.7%	92.6%	91.3%
hu_stair-up	73.2%	78.9%	89.1%	89.3%
hs_stair-up	78.3%	81.1%	89.5%	92.3%
hu_stair-down	79.6%	85.6%	92.2%	91.1%
hs_stair-down	86.7%	86.7%	92.4%	93.9%

**Table 9:** Confusion matrix of multi-floor motion recognition using original RF with PCR2&5.

Classified as→	a	b	c	d	e	f	g	h	i	Total
a = hu_static	416	6	3	3	2	6	0	0	0	436
b = hu_elevator-up	6	80	0	0	0	3	0	0	0	89
c = hu_elevator-down	7	0	77	0	0	0	0	0	0	84
d = hu_walk	7	0	0	270	0	3	0	4	0	284
e = hs_walk	0	0	0	0	428	0	7	0	6	441
f = hu_stair-up	3	0	0	17	0	140	0	1	0	161
g = hs_stair-up	0	0	0	0	13	0	122	0	0	135
h = hu_stair-down	9	0	0	13	0	0	0	103	0	125
i = hs_stair-down	0	0	0	3	17	0	2	0	124	146
Total	448	86	80	306	460	152	131	108	130	1901

**Table 10:** Confusion matrix of multi-floor motion recognition using improved RF with PCR2/5.

Classified as→	a	b	c	d	e	f	g	h	i	Total
a = hu_static	419	3	5	1	4	3	0	1	0	436
b = hu_elevator-up	4	81	0	0	0	2	2	0	0	89
c = hu_elevator-down	2	0	80	0	0	0	0	2	0	84
d = hu_walk	3	0	0	272	0	3	0	6	0	284
e = hs_walk	1	0	0	0	438	0	5	0	2	446
f = hu_stair-up	1	3	0	6	0	153	0	2	0	165
g = hs_stair-up	0	0	0	0	4	0	124	0	2	130
h = hu_stair-down	6	0	0	3	3	0	0	109	0	121
i = hs_stair-down	0	0	0	3	8	0	4	0	131	146
Total	436	87	85	285	457	161	135	120	135	1901

## Abbreviations

The following abbreviations are used in this manuscript:

GPS	Global Positioning System
AGM	accelerometer, gyroscope and magnetometer
H-motions	stay static or move on one floor
V-motions	move with vertical displacement
RF	Random Forest
J48 DT	J48 Decision Tree
ANN	Artificial Neural Network
NB	Native Bayes
SVM	Support Vector Machine
